# scRNASeqDB: A Database for RNA-Seq Based Gene Expression Profiles in Human Single Cells

**DOI:** 10.3390/genes8120368

**Published:** 2017-12-05

**Authors:** Yuan Cao, Junjie Zhu, Peilin Jia, Zhongming Zhao

**Affiliations:** 1 Center for Precision Health, School of Biomedical Informatics, The University of Texas Health Science Center at Houston, Houston, TX 77030, USA; yuan.cao@uth.tmc.edu (Y.C.); Peilin.Jia@uth.tmc.edu (P.J.); 2 Department of Electrical Engineering, Stanford University, Stanford, CA 94305, USA; jjzhu@stanford.edu; 3 Human Genetics Center, School of Public Health, The University of Texas Health Science Center at Houston, Houston, TX 77030, USA; 4 Department of Biomedical Informatics, Vanderbilt University Medical Center, Nashville, TN 37203, USA

**Keywords:** single cell, RNA sequencing, database, expression profile, cell type, differential expression

## Abstract

Single-cell RNA sequencing (scRNA-Seq) is rapidly becoming a powerful tool for high-throughput transcriptomic analysis of cell states and dynamics at the single cell level. Both the number and quality of scRNA-Seq datasets have dramatically increased recently. A database that can comprehensively collect, curate, and compare expression features of scRNA-Seq data in humans has not yet been built. Here, we present scRNASeqDB, a database that includes almost all the currently available human single cell transcriptome datasets (*n* = 38) covering 200 human cell lines or cell types and 13,440 samples. Our online web interface allows users to rank the expression profiles of the genes of interest across different cell types. It also provides tools to query and visualize data, including Gene Ontology and pathway annotations for differentially expressed genes between cell types or groups. The scRNASeqDB is a useful resource for single cell transcriptional studies. This database is publicly available at https://bioinfo.uth.edu/scrnaseqdb/.

## 1. Introduction

Single-cell RNA sequencing (scRNA-Seq) is rapidly evolving as a powerful tool for high-throughput transcriptomic analysis of cell states and dynamics [1]. So far, most gene expression studies in the literature have been based on the averaged expression information from bulk tissue [2]. scRNA-Seq can accurately measure RNA expression characteristics at the single cell level in order to explore cell phenotype, function, genetic alterations in various conditions, and transcriptomic heterogeneity. While it is a new technology, both the quality and quantity of scRNA-Seq data have dramatically increased during the past a few years [3,4,5,6,7,8,9]. Such an overwhelming volume of public scRNA-Seq data requires effective quality control, platform and analysis assessment, data mining and integration, and information management. Therefore, there is strong need for a centralized web resource that curates and provides features such as single-cell gene expression profiles among various cell types and studies. Such a resource will be beneficial to the broad biological and biomedical research communities.

So far, there have been three reported web resources for single cell transcriptome data, but all of them were developed for mouse data [10,11,12]. Du et al. [10] developed a web resource which only included one single-cell gene expression dataset that was involved in the development of mouse lung tissue. Similarly, Nestorowa et al. [11] built a web interface using only one single-cell transcriptome dataset for hematopoietic stem and progenitor cells (HPSC), providing insights into the differentiation of blood stem cell in mice. Finally, Biase et al. [12] developed an online genome browser for mouse scRNA-Seq data. Recently, the single-cell portal for brain research was launched by the Broad Institute of MIT and Harvard (https://portals.broadinstitute.org/single_cell). This portal was developed to facilitate sharing scientific results and disseminate datasets resulting from the National Institutes of Health (NIH) Brain Research through Advancing Innovative Neurotechnologies (BRAIN) initiative, which contains mostly brain related scRNA-Seq datasets in humans and mice. To date, it includes 39 studies covering 234,551 cells. However, there are only 15 shared datasets that are freely accessible, and they contain no more than 20 human cell lines or cell types. Although there are some databases that offer abundant transcriptomic information for one or multiple cell lines or cell types, an in-depth investigation of transcriptional features and data comparison of the transcriptomes across different studies in humans are still lacking [13,14].

To the best of our knowledge, there is no dedicated database for the comprehensive curation and annotation of human scRNA-Seq data yet. Here, we developed human single-cell RNA-Seq database (scRNASeqDB), a database that collects and curates publicly available single cell gene expression datasets for humans. Through metadata annotation and unified data processing procedures, it covers 200 human cell groups and 13,440 samples (as of 1 September 2016). These data and the follow-up analysis results are available through a user-friendly web interface. First, scRNASeqDB allows ranking gene expression across different datasets; that is, the user can analyze a specific gene across different cell lines and cell types. Second, based on the transcriptional features of each cell group in one dataset, we developed a visualization interface that allows the flexible display and comparison of user-specified gene(s). Third, scRNASeqDB provides users with the gene rank list for a specific cell group. This database is publicly available at https://bioinfo.uth.edu/scrnaseqdb/. It helps researchers within the fields of biology and medicine to facilitate gene expression studies in human single cells.

## 2. Materials and Methods

### 2.1. Data Collection and Metadata Annotations

We searched the National Center for Biotechnology Information (NCBI) Gene Expression Omnibus (GEO) database [15] for gene expression profiling experiments using the following keywords: scRNAseq, single-cell RNA-Seq, and single-cell transcriptome. Such strategies have been successfully utilized in our previous work and in a wide range of databases developed by our group [16,17,18] and by others [19,20,21,22]. A detailed description is provided in Table S1. Next, we carefully reviewed the resultant papers and datasets. A total of 38 datasets for human single-cell RNA-Seq analysis was obtained from this systematic search (Table S2). The number of samples ranged between 7 and 3162. The number of scRNA-Seq accessible experiments in humans has increased dramatically since 2012, with 2016 alone contributing 5348 samples. These datasets included 200 human cell groups, which are related to reproduction, immune system, the brain and nervous system, cancer, and stem cells, among others (Table S3). The metadata of GEO datasets were downloaded and imported into MySQL using the R package GEOquery [23] and RMySQL, respectively. Metadata of cell types and experiment conditions were manually curated according to the characteristics of each dataset or description in the original publications. For RNA-Seq experiments, the gene expression matrices were also retrieved from the GEO and converted to Transcripts Per Million (TPM) or read count format by using our in-house R scripts (available upon request). For cells in datasets where the fragments per kilobase of exon per million reads mapped (FPKM) were available, we computed the TPM for gene *i*, according to:

(1) $${\mathrm{TPM}}_{i}=\left(\frac{{\mathrm{FPKM}}_{i}}{\sum_{j}{\mathrm{FPKM}}_{j}}\right)\times 10^{6}$$

For datasets that provided expression data with unique molecular identifier (UMI) values instead of FPKM values, we similarly applied the same normalization as we did for TPM calculation for each cell. This conversion enables the units to be consistent for dataset-to-dataset comparison.

In addition, the sample accession number for each sample was added, which uniquely identifies the exact experiment in the NCBI GEO dataset [24].

### 2.2. Rank of Genes

RankProd is a non-parametric analysis tool that employs the rank of the expression value of genes to prioritize those genes [25]. In scRNASeqDB, RankProd was adopted to compare gene expression level across different scRNA-Seq datasets. Each gene was assigned an order in the specific cell lines or cell types. A low gene order means it is highly expressed in the cell line or cell type of the dataset in examination. In our database, we used a horizontal bar plot to represent a gene’s rank in each cell line or cell type. Specifically, we defined the rank of a gene in a particular cell line or cell type as follows:

(2) $$\frac{\mathrm{total}\;\mathrm{gene}\;\mathrm{number}-\mathrm{gene}\;\mathrm{order}\;}{\mathrm{total}\;\mathrm{gene}\;\mathrm{number}}\times 100$$

For each gene, its rank in all datasets was presented using a bar plot, providing the users with an overview and direct comparison of the gene’s expression rank across the different datasets.

### 2.3. Differential Gene Expression

For particular studies that contained more than one cell line or experimental condition, such as those that included pancreatic islet acinar versus pancreatic islet duct [26], or those that included iPSC 409B2 (41 days) versus iPSC 409B2 (65 days), we conducted differentially expressed (DE) gene analysis between cell groups for each dataset. BPSC, representing Beta-Poisson model for Single-Cell RNA-seq data analyses, [27] was employed to analyze the DE genes. BPSC is an analysis tool based on the beta-Poisson model for single-cell gene expression data. It addresses practical and realistic issues such as non-integer expression values or low expression values. Utilizing BPSC, we analyzed DE genes in all 38 datasets by parallel computing in a server with 16 CPUs and 256 GB RAM. The total running time was 25.7 days. The genes were identified as significantly differentially expressed at a false discovery rate (FDR) <0.05 [28]. Finally, we deposited these DE genes into our database and made them available to users at the bottom section in ‘Dataset View’.

### 2.4. Web Interface

Currently, scRNASeqDB release 1.0 has collected 13,440 samples belonging to 200 human cell groups from 38 datasets. The web interface of scRNASeqDB was implemented in PHP and JavaScript using the Yii framework, which enables users to search across the database easily without requiring much computer expertise. Interactive heatmap and box plots were constructed dynamically to display gene expression for individual cells and cell groups in one dataset using the HighCharts component. Users can export the charts as images in PDF, PNG, JPG, or SVG format for record or further analysis.

## 3. Results

### 3.1. Database Description and Summary of Features

**Figure 1:**
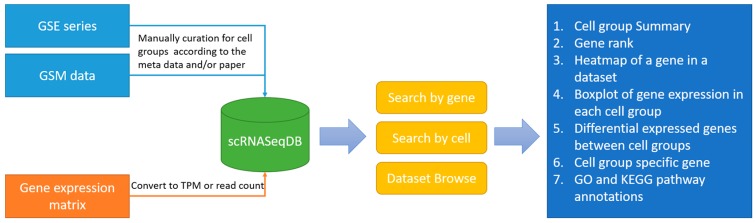
Flowchart of data collection and database construction. GSE: accession number of series; GSM: accession number for samples; TPM: Transcripts Per Million.

**Figure 2:**
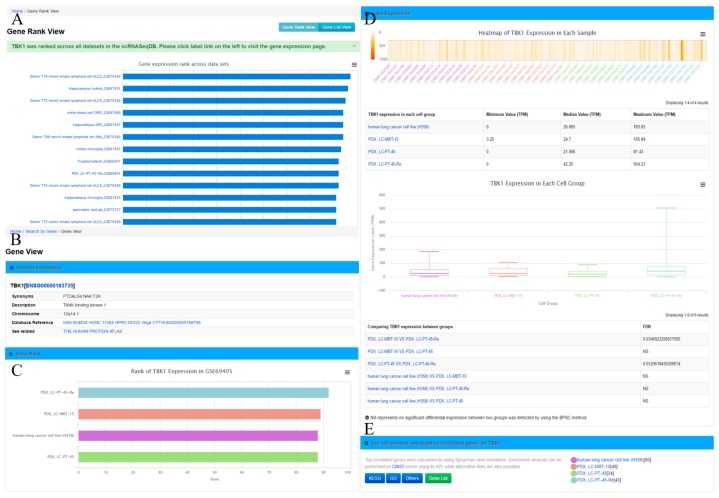
Gene Rank View and Gene View. (**A**) Gene rank list of an example gene *TBK1* across all datasets; (**B**) Display general information of *TBK1* in ‘Gene View’ page; (**C**) Show the gene rank of *TBK1* in each cell group of GSE69405; (**D**) The section of gene expression displayed by heatmap and box plots. The minimum value, median value, and maximum value of the gene expression and the FDR (false discovery rate) of the gene expression comparison between groups are also displayed; (**E**) The top 100 positively and negatively correlated genes and annotation links are provided.

The main purpose of scRNASeqDB is to facilitate the analysis and visualization of gene expression profiles across various human single cells based on the public datasets. The flowchart of data collection and database construction is provided in Figure 1. We have collected 38 single-cell RNA-seq datasets of humans covering 200 cell groups in the scRNASeqDB. The homepage of the web resource provides three ways to search the database. First, the user can search the gene of interest using gene symbols or gene Ensembl IDs. A successful search will lead the user to the ‘Gene Rank List’ webpage (Figure 2A), which displays the gene rank across different cell lines or types in this database. The ‘Gene View’ page provides detailed information of the gene expression within the dataset (Figure 2B–E). Second, the user can search the cell group of interest by inputting the name of a cell line or type. As an outcome, it will generate a ‘Cell View’ webpage with rich information. Finally, the user can browse the datasets by choosing a dataset ID; the system will generate a ‘Dataset View’ webpage. The user can also hit the tags of some popular genes in the ‘Gene Cloud’ section to start a quick gene search.

The ‘Gene View’ page displays the expression profile of a gene. It consists of four sections. (1) The general information section includes gene symbol, synonyms, Ensembl gene ID, description, and chromosome locations, among others. To further enable the exploration of gene information, this section provides web links of the gene to other online resources, such as OMIM [29], Ensembl [30], HPRD [31], and Vega [32]; (2) In the gene rank section, a bar plot shows the specific gene rank across different cell types/groups in this study; (3) In the gene expression section, an interactive heatmap is available to display gene expression across individual cells in one dataset. A box plot and a table summarizing the minimum, median, and maximum value of the gene expression in each cell group from each dataset is also provided. If there are more than one cell group in a dataset, a comparison of query gene expression among cell groups will also be listed to indicate significantly differentially expressed genes; (4) It also shows the top 100 positively and negatively correlated genes across cells in a dataset for the query gene. Pathway annotations from Gene Ontology (GO) and Kyoto Encyclopedia of Genes and Genomes (KEGG) [33] for these genes are provided as well. In addition, the user can obtain the top 100 positively and negatively correlated genes by clicking the ‘Gene List’ button for further analysis.

**Figure 3:**
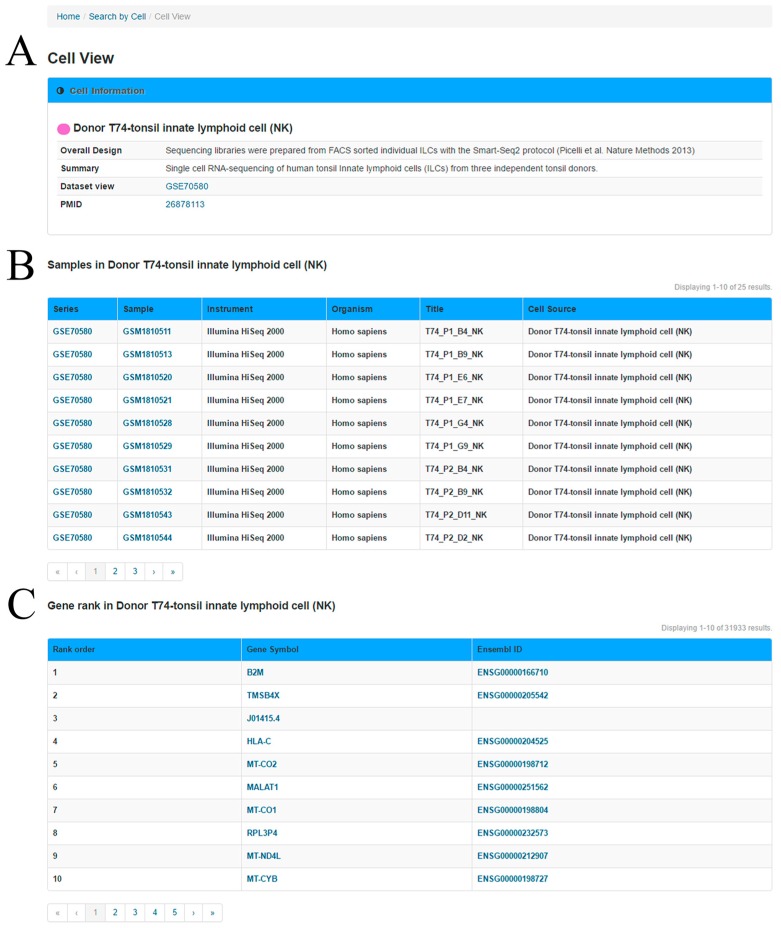
Cell View, displaying tonsil innate lymphoid cell (NK) information as an example. (**A**) Summary information; (**B**) Metadata of cell samples; (**C**) The top 200 upregulated genes and correlation matrices for these genes for the specific cell type or group. Note that multiple pages will be displayed when the sample or gene list is long.

In the ‘Cell View’ webpage (Figure 3), the user can explore the ranked genes in the query cell group and the relationship between two genes in this specific cell group. In the first section, a summary of the cells and experiments are provided based on the metadata of the GEO. In the second section, a table is used to present the information of cell samples in the cell group.

**Figure 4:**
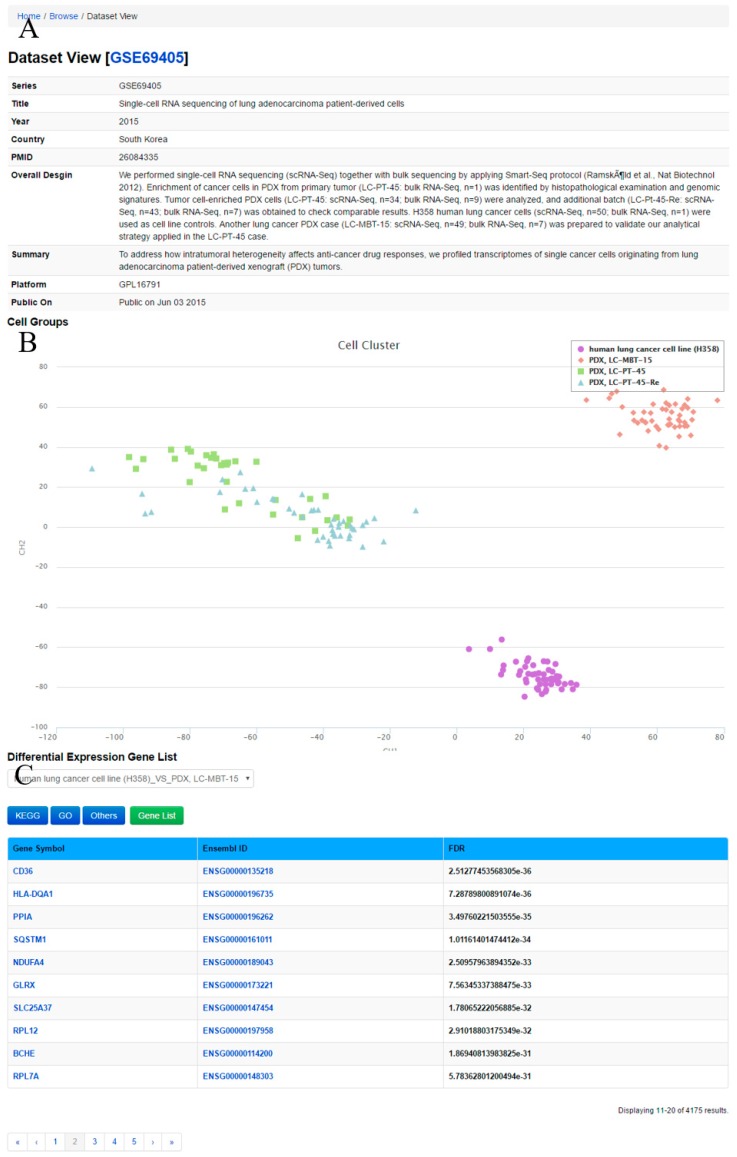
Dataset View, displaying dataset GSE69405 as an example. (**A**) Metadata information for the dataset; (**B**) Cell groups and cell cluster are presented; (**C**) A list of differentially expressed genes between different groups is provided.

Through ‘Dataset View’ (Figure 4), the user can obtain a comprehensive description and cell groups in the query dataset. The method Clustering through Imputation and Dimensionality Reduction (CIDR) [34] is used to construct cell clusters based on the datasets. Meanwhile, lists of differentially expressed genes between two cell groups in the datasets as well as GO and KEGG pathway annotations for differentially expressed genes are displayed.

### 3.2. Example

Many genes demonstrate a tissue-specific expression profile [24,35]. For example, Leucine-rich-alpha-2-glycoprotein1 (LRG1) is encoded by an oncogene that was recently found to be vital to the progression of human cancer [36]. *LRG1* mRNA level is upregulated in most hepatocellular carcinoma (HCC) cell lines [36]. Searching *LRG1* expression in scRNASeqDB confirmed similar expression in single cells, with the highest expression rank (top 4% rank) observed in liver cancer cells (Figure S1). This example indicates that scRNASeqDB can be used as a tool to identify tissue-selective genes in a single cell.

## 4. Discussion

scRNASeqDB is a user-friendly database that timely collects and curates single cell gene expression profiles in human cells. The database currently includes 38 datasets covering the gene expression of 13,440 single cells from 200 cell groups. It provides various features such as gene expression in different cell types, expression patterns at the pathway levels, and tools for the visualization and exploration of gene expression in single cells. In order to provide investigators with a tool to accurately determine under which condition(s) and in which cell line(s) and cell type(s) the expression of a gene of interest is altered, we developed analysis tools for the study of the dynamics of gene expression regulation across cell lines and types. The online results can be easily saved in different file formats for other purposes. Furthermore, scRNASeqDB was designed to facilitate the identification of specific gene markers for each cell subpopulation by using differential gene analysis.

A common task in many single-cell studies is to detect differentially expressed genes between cell populations [37]. To the best of our knowledge, there are some methods that are designed specifically for scRNA-Seq data, such as scDD (a statistical approach for identifying differential distributions in single-cell RNA-seq experiments) [38], D3E (discrete distributional differential expression) [39], MAST (Model-based Analysis of Single-cell Transcriptomics) [40], SCDE (a set of statistical methods for analyzing single-cell RNA-seq data) [41], and BPSC [27]. In Jaakkola’s method evaluation [37], the authors reported that ROTS (reproducibility-optimized test statistic) [42] had the best performance after they compared SCDE, MAST, DESeq, Limma, and ROTS using three benchmark datasets. In our other study, we compared the performance across several tools in scRNA-Seq differential expression analysis. Although scDD showed better performance in some datasets, it took much more time when the sample size is not small (unpublished data). BPSC shows comparable performance with higher sensitivity, specificity, and reproducibility, as well as being less time-consuming. Therefore, we employed BPSC for DE analysis in our database.

With the rapid evolution of single-cell sequencing technologies, we expect an even faster generation of scRNA-Seq data in the next a few years. We plan to update the database by following our in-house pipeline. Updated keywords representing scRNA-Seq studies will be incorporated to include newly arising datasets. Our pipeline will also be continuously updated to keep with the development of new data formats or data types. In addition, we provide function to allow users to submit their own single-cell RNA-seq data. The user can conduct this task by submitting the request for review to the web administrator through our web interface. Upon approval, the dataset along with related information will be imported to this database. Such data, along with the data we retrieve from public domains like GEO, will be analyzed in our future database release. Second, gene expression dataset from relevant bulk tissue will be integrated for the assessment of expression features and biases between scRNA-Seq and traditional RNA-Seq. Third, a Gene Set Enrichment Analysis (GSEA) module will be integrated into scRNASeqDB to allow the user to perform advanced gene feature analysis based on the scRNA-Seq data. In addition to these future expansions, our database will provide an overview of the features of gene expression in various single cells, providing useful and general information on expression in different cell lines and tissues. Thus, when data eventually increase to beyond our maintenance capabilities and become sufficient for a general resource of scRNA-Seq for representative cell types and tissues, we believe that we will have accomplished our endeavor for such purposes and conclude our development of this database.

## 5. Conclusions

In summary, we have created scRNASeqDB to allow users to search, analyze, compare, and visualize the gene expression from most available scRNA-Seq data so that they can seek deeper insights into gene expression across human single cells. scRNASeqDB will be regularly maintained to include future scRNA-Seq datasets, both from the user’s direct submission to the scRNASeqDB and from the annotation of scRNA-Seq data from public resources such as GEO. More functions will be added to our database’s web interface as well. So far, this is the first single-cell RNASeq database for humans.

## Supplementary Materials

The following are available online at www.mdpi.com/2073-4425/8/12/368/s1. Figure S1: *LRG1* gene expression rank across scRNA-Seq datasets. The figure was generated by searching *LRG1* from scRNASeqDB and saved in one of the available formats (PDF, PNG, JPG, or SVG). Table S1: Summary of the search results in the PubMed database by different search strategies (by 1 July 2016). Table S2: Summary of datasets in scRNASeqDB. Table S3: Summary of cell lines or types in scRNASeqDB.
