# Supplementary material for: scRNASeqDB: A Database for RNA-Seq Based Gene Expression Profiles in Human Single Cells
Source: Genes (Basel). 2017 Dec 5;8(12):368. doi: 10.3390/genes8120368 (PMC5748686; doi:10.3390/genes8120368)

Gene expression rank across data sets

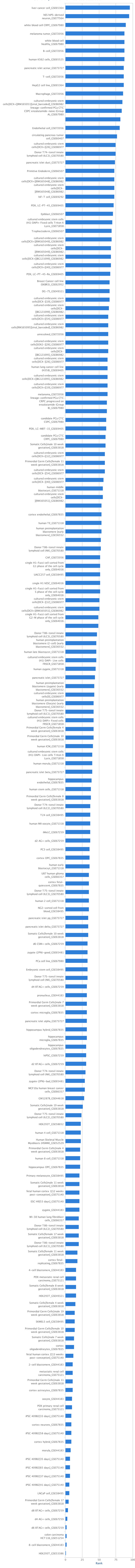

Supplement: Supplementary file 1 [file scRNASeqDB Suppl Figure S1.pdf › scRNASeqDB Suppl Figure S1.pdf]
