# Supplementary material for: scRNASeqDB: A Database for RNA-Seq Based Gene Expression Profiles in Human Single Cells
Source: Genes (Basel). 2017 Dec 5;8(12):368. doi: 10.3390/genes8120368 (PMC5748686; doi:10.3390/genes8120368)
Supplement: Supplementary file 1 [file genes-08-00368-s001.zip › scRNASeqDB Suppl Tables S1-3.pdf]

Supplementary Tables

Table S1. Summary of the search results in the PubMed database by different search strategies (by July 1st, 2016)

Search Number	Search phrase	# papers
#1	(single cell[Title/Abstract]) AND (transcriptome[Title/Abstract])	293
#2	(single cell[Title/Abstract]) AND (RNA-seq[Title/Abstract])	234
#3	scRNA-seq	11
#4	scRNA seq	26
#5	single-cell rna-seq	191
#6	single-cell transcriptomics	129
#7	single-cell rna-sequencing	497
#8	#1 U #2 U #3 U #4 U #5 U #6 U #7*	653
#9	Keep human rna-seq GEO data sets based on #8	36

* U indicates the union between two sets.

Table S2 Summary of datasets in scRNASeqDB

Dataset ID	Title	PMID	Year	Cell group [cell number]	Platform
GSE36552	Tracing pluripotency of human early embryos and embryonic stem cells by single cell RNA-seq	23934149	2013	human embryonic stem cell [peri-implantation stem cells][34]<br>human preimplantation blastomere (2-cell) [early blastomere][6]<br>human preimplantation blastomere (Oocyte) [early blastomere][3]<br>human preimplantation blastomere (zygote) [early blastomere][3]<br>human preimplantation blastomere [early blastomere][78]	GPL11154
GSE38495	Full-length mRNA-Seq from single-cell levels of RNA and individual circulating tumor cells	22820318	2012	Embryonic stem cell[8]<br>LNCaP cell[6]<br>NG2-sorted cell from blood[6]<br>PC3 cell[4]<br>Primary melanocyte[2]<br>SKMEL5 cell[4]<br>T24 cell[4]<br>UACC257 cell[3]	GPL10999<br>GPL11002<br>GPL13112<br>GPL15520

GSE44183	Genetic Programs in Human and Mouse Early Embryos Revealed by Single-Cell RNA-Sequencing	23892778	2013	2-cell blastomere[3]<br>4-cell blastomere[4]<br>8-cell blastomere[10]<br>morula[3]<br>oocyte[3]<br>pronucleus[3]<br>zygote[2]	GPL11154<br>GPL13112
GSE44618	Single Cell RNA-Seq	24299736	2013	GM12878[28]	GPL11154
GSE49321	Improved Smart-Seq for sensitive full-length transcriptome profiling in single cells	24056875	2013	DG-75[7]<br>HEK293T[39]	GPL11154<br>GPL13112
GSE51254	Quantitative assessment of single-cell RNA sequencing methods	24141493	2013	colon carcinoma HCT116[109]	GPL11154
GSE52529	Pseudo-temporal ordering of individual cells reveals regulators of differentiation	24658644	2014	Human Skeletal Muscle Myoblasts (HSMM)[372]	GPL11154<br>GPL16791
GSE53386	Single-cell RNA-Seq transcriptome analysis of circular RNAs in mouse embryos	NA	2015	HEK293T[10]	GPL11154<br>GPL13112<br>GPL16791<br>GPL17021
GSE57872	Single cell RNA-seq of primary human glioblastomas	24925914	2014	glioblastoma [Cla][45]<br>glioblastoma [Cla+Mes][6]<br>glioblastoma [Mes][101]<br>glioblastoma [Neu][24]<br>glioblastoma [Neu+Cla][2]<br>glioblastoma [Neu+Mes][19]<br>glioblastoma [None][120]<br>glioblastoma [Pro][88]<br>glioblastoma [Pro+Cla][20]<br>glioblastoma [Pro+Neu][5]	GPL16791

GSE58652	Tn5 transposase and tagmentation procedures for massively scaled sequencing projects	25079858	2014	HEK293T[42]	GPL11154 GPL13112 GPL15228
GSE60407	Human Pancreatic CTCs Express the ECM Protein SPARC	25242334	2014	circulating pancreas tumor cell[7]	GPL16288
GSE62952	Integrated genome and transcriptome sequencing from the same cell	25599178	2014	Breast Cancer cell line (SKBR3)[21]	GPL16791 GPL17021

GSE63818	The Transcriptome and DNA Methylome Landscapes of Human Primordial Germ Cells	26046443	2015	Primordial Germ Cells[female 10 week gestation][13]<br>Primordial Germ Cells[female 11 week gestation][10]<br>Primordial Germ Cells[female 17 week gestation][31]<br>Primordial Germ Cells[female 4 week gestation][12]<br>Primordial Germ Cells[female 8 week gestation][27]<br>Primordial Germ Cells[male 10 week gestation][19]<br>Primordial Germ Cells[male 11 week gestation][27]<br>Primordial Germ Cells[male 19 week gestation][57]<br>Primordial Germ Cells[male 4 week gestation][6]<br>Primordial Germ Cells[male 7 week gestation][40]<br>Somatic Cells[female 10 week gestation][3]<br>Somatic Cells[female 11 week gestation][10]<br>Somatic Cells[female 17 week gestation][9]<br>Somatic Cells[female 4 week gestation][6]<br>Somatic Cells[female 8 week gestation][10]<br>Somatic Cells[male 10 week gestation][11]<br>Somatic Cells[male 11 week gestation][8]<br>Somatic Cells[male 19 week gestation][3]<br>Somatic Cells[male 7 week gestation][26]	GPL11154<br>GPL16791
GSE64016	Single cell RNA-seq data of human hESCs to evaluate Oscope - a statistical pipeline for identifying oscillatory genes in unsynchronized single cell RNA-Seq	26301841	2015	single H1 hESC[213]<br>single H1-Fucci cell sorted from G1 phase of the cell cycle only[91]<br>single H1-Fucci cell sorted from G2-M phase of the cell cycle only[76]<br>single H1-Fucci cell sorted from S phase of the cell cycle only[80]	GPL16791

GSE65364	Single Cell triple omics sequencing reveals genetic and epigenetic heterogeneity in hepatocellular carcinoma	26902283	2016	HepG2 cell line[8]<br>liver cancer cell[26]	GPL11154<br>GPL16791<br>GPL17021<br>GPL20795
GSE65481	Human Embryo Single Cell Transcriptome Analysis	26904963	2015	zygote (2PN)-bad[11]<br>zygote (2PN)-good[11]	GPL11154
GSE65525	Droplet barcoding for single cell transcriptomics applied to embryonic stem cells	26000487	2015	human K562 cells[239]	GPL16417<br>GPL16791<br>GPL17021<br>GPL18573<br>GPL19057
GSE66053	Single mammalian cells compensate for differences in cellular volume and DNA copy number through independent global transcriptional mechanisms	25866248	2015	primary foreskin fibroblast cell CRL2097 (fixed cell)[14]<br>primary foreskin fibroblast cell CRL2097 (large live)[74]<br>primary foreskin fibroblast cell CRL2097 (med live)[3]	GPL16791<br>GPL18573
GSE66357	Scalable Microfluidics for Single Cell RNA Printing and Sequencing	26047807	2015	MCF10a human breast cancer cells[102]<br>U87 human glioma cells[138]<br>WI-38 human lung fibroblast cells[45]	GPL18573
GSE66507	Single-Cell RNA-seq Defines the Three Cell Lineages of the Human Blastocyst	26293300	2015	Epiblast[8]<br>Primitive Endoderm[6]<br>Trophectoderm[16]	GPL11154

GSE67259	Robust In Vitro Induction of Human Germ Cell Fate from Pluripotent Stem Cells	26189426	2015	d2 AG+ cells[2]<br>d2 BTAG+ cells[5]<br>d4 AG+ cells[2]<br>d4 BTAG+ cells[5]<br>d6 BTAG+ cells[4]<br>d6 CSM+ cells[2]<br>d8 BTAG+ cells[2]<br>hiPSC[11]<br>iMeLC[4]	GPL15907<br>GPL16288<br>GPL19944
GSE67835	A survey of human brain transcriptome diversity at the single cell level	26060301	2015	cortex astrocytes[62]<br>cortex endothelial[17]<br>cortex fetal-quiescent[110]<br>cortex fetal-replicating[25]<br>cortex hybrid[44]<br>cortex microglia[8]<br>cortex neurons[130]<br>cortex oligodendrocytes[32]<br>cortex OPC[2]<br>hippocampus endothelial[3]<br>hippocampus hybrid[2]<br>hippocampus microglia[8]<br>hippocampus neurons[1]<br>hippocampus oligodendrocytes[6]<br>hippocampus OPC[16]	GPL15520<br>GPL18573
GSE67980	RNA-Seq of Single Prostate CTCs Implicates Non-Canonical Wnt Signaling in Antiandrogen Resistance	26383955	2015	candidate PCa CTC CRPC[33]<br>candidate PCa CTC CSPC[12]<br>lineage-confirmed PCa CTC CRPC enzalutamide-naive (Group A)[37]<br>lineage-confirmed PCa CTC CRPC progressed on enzalutamide (Group B)[36]<br>lineage-confirmed PCa CTC CSPC enzalutamide-naive (Group A)[4]<br>PCa cell line[30]<br>white blood cell CRPC[2]<br>white blood cell healthy[3]	GPL16288
GSE68596	Genome-wide maps of nuclear lamina interactions in single human cells (CEL-seq)	26365489	2015	Cell line KBM7, derived from a patient with chronic myeloid leukemia[96]	GPL16791

GSE69292	Integrative analyses of human reprogramming reveal dynamic nature of induced pluripotency [smartseq2]	26186193	2015	hiF-T cell[52]	GPL16791
GSE69405	Single-cell RNA sequencing of lung adenocarcinoma patient-derived cells	26084335	2015	human lung cancer cell line (H358)[50]<br>PDX, LC-MBT-15[49]<br>PDX, LC-PT-45[34]<br>PDX, LC-PT-45-Re[43]	GPL16791
GSE69790	Single cell transcriptomics analysis of induced pluripotent stem cell-derived cortical neurons reveals frequent dual layer identity	26740550	2016	iPSC-derived cortical neuron[16]	GPL11154

GSE70580	Single cell RNA-sequencing of human tonsil Innate lymphoid cells (ILCs)	26878113	2016	Donor T74-tonsil innate lymphoid cell (ILC1)[1]<br>Donor T74-tonsil innate lymphoid cell (ILC2)[11]<br>Donor T74-tonsil innate lymphoid cell (ILC3)[38]<br>Donor T74-tonsil innate lymphoid cell (NK)[25]<br>Donor T75-tonsil innate lymphoid cell (ILC1)[60]<br>Donor T75-tonsil innate lymphoid cell (ILC2)[62]<br>Donor T75-tonsil innate lymphoid cell (ILC3)[128]<br>Donor T75-tonsil innate lymphoid cell (NK)[23]<br>Donor T86-tonsil innate lymphoid cell (ILC1)[66]<br>Donor T86-tonsil innate lymphoid cell (ILC2)[66]<br>Donor T86-tonsil innate lymphoid cell (ILC3)[142]<br>Donor T86-tonsil innate lymphoid cell (NK)[26]	GPL11154
GSE71315	Single cell analysis of long non-coding RNAs in the developing human neocortex	27081004	2016	Cerebral Cortex GW19.5[26]<br>Cerebral Cortex GW20.5[123]<br>Cerebral Cortex GW23.5[77]	GPL16791
GSE71318	Tracing the expression of circular RNAs in human preimplantation embryos	NA	2016	human 2 cell[4]<br>human 4 cell[4]<br>human 8 cell[5]<br>human early blastocsyt[3]<br>human ICM[3]<br>human late blastoycst[3]<br>human middle blastocyst[3]<br>human MII oocyte[4]<br>human morula[4]<br>human stem cells[7]<br>human TE[3]<br>human zygote[5]	GPL16791

GSE71858	Fixed single-cell transcriptomic characterization of human radial glial diversity	26524239	2015	cultured embryonic stem cells (H1) DAPI- Live cells FRSCR[15]<br>cultured embryonic stem cells (H1) DAPI- Live cells Triton X Lysis[11]<br>cultured embryonic stem cells (H1) DAPI+ Fixed cells FRSCR[12]<br>cultured embryonic stem cells (H1) DAPI+ Fixed cells Triton X Lysis[7]	GPL16791
GSE72056	Single cell RNA-seq analysis of melanoma	27124452	2016	B-cell[326]<br>CAF[39]<br>Endothelial cell[40]<br>Macrophage[42]<br>melanoma[1071]<br>melanoma tumor[246]<br>T-cell[1302]<br>unresolved[96]	GPL18573
GSE73121	Single-cell transcriptome profiling for metastatic renal cell carcinoma patient-derived cells [RNA-seq]	27139883	2015	metastatic renal cell carcinoma[35]<br>PDX metastatic renal cell carcinoma[36]<br>PDX primary renal cell carcinoma[47]	GPL16791
GSE73727	Single-cell transcriptomics reveals unique features of human pancreatic islet cell subtypes	26691212	2016	pancreatic islet[4]<br>pancreatic islet acinar[11]<br>pancreatic islet alpha[18]<br>pancreatic islet beta[12]<br>pancreatic islet delta[2]<br>pancreatic islet duct[8]<br>pancreatic islet pp[9]	GPL11154
GSE75140	Human cerebral organoids recapitulate gene expression programs of fetal neocortex development.	26644564	2015	ESC H9[53 days][96]<br>fetal human cortex 1[12 weeks post-conception][164]<br>fetal human cortex 2[13 weeks post-conception][62]<br>iPSC 409B2[33 days][40]<br>iPSC 409B2[35 days][68]<br>iPSC 409B2[37 days][71]<br>iPSC 409B2[41 days][74]<br>iPSC 409B2[58 days][79]<br>iPSC 409B2[65 days][80]	GPL16791

GSE77564	Coupled electrophysiological recording and single-cell transcriptome analyses revealed molecular mechanisms underlying neuronal maturation	26883038	2016	hES hiPS-derived neuron[20]	GPL16791

Table S3. Summary of cell lines or types in scRNASeqDB

**Category**	**Cell line or cell type**
Reproduction relate	blastomere
	zygote
	oocyte
	morula
	trophectoderm
	somatic cells from gestation
	pronucleus
	primordial germ cell (PGC)
Immune related	T-cell
	B-cell
	white blood cell (WBC)
	B lymphocyte cell line (DG-75)
	tonsil innate lymphoid cell (ILC)
	tonsil innate lymphoid cell (NK)
	lymphoblastoid cell line (GM12878)
	hiF-T cell
	macrophage
Cancer related	HepG2 cell line
	liver cancer cell
	breast cancer cell line (SKBR3)
	breast cancer cell line (MCF10a)
	human melanoma cell line (UACC257)
	primary melanocyte
	melanoma cell line (SK-MEL-5)
	melanoma cell
	human glioma cell line (U87)
	glioblastoma cell
	metastatic renal cell carcinoma

	PDX metastatic renal cell carcinoma
	PDX primary renal cell carcinoma
	human lung cancer cell line (H358)
	PDX, lung cancer
	human lung normal fibroblast cell line (WI-38)
	cancer associated fibroblasts (CAFs)
	cell line KBM7, derived from a patient with chronic myeloid leukemia
	urinary bladder transitional cell carcinoma cell line (T24)
	prostate carcinoma cell line (LNCaP)
	PC3 cell line
	prostate cell line
	prostate circulating tumor cell
	pancreas circulating tumor cell
	pancreatic islet
	colon carcinoma cell line (HCT116)
The brain and nervous system	cerebral cortex
	cortex astrocytes
	cortex endothelial
	cortex fetal-quiescent
	cortex fetal-replicating
	cortex hybrid
	cortex neurons
	cortex oligodendrocytes
	cortex OPC
	fetal human cortex
	hES hiPS-derived neuron
	iPSC-derived cortical neuron
	hippocampus endothelial
	hippocampus hybrid
	hippocampus microglia
	hippocampus neurons
	hippocampus oligodendrocytes
	hippocampus OPC
Stem cell related	human K562 cells
	human embryonic stem cell (hESC)
	human induced pluripotent stem cell (hiPSC)
	iPSC cell line 409B2
Others	endothelial cell
	epiblast cell
	HEK293T
	primitive endoderm

	primary foreskin fibroblast cell (CRL2097)
	human skeletal muscle myoblasts (HSMM)
